# Crystal structures of two polymorphs of tixocortol pivalate

**DOI:** 10.1107/S2056989021007167

**Published:** 2021-07-16

**Authors:** Yoann Rousselin, Sylvie Yolka, Alexandre Clavel

**Affiliations:** a ICMUB - UMR 6302, Université Bourgogne Franche Comte, 9 avenue Alain Savary, 21000 DIJON, France; b M2i Salin, 36 Route Arles RD 36, 13129 Salin de Giraud, France

**Keywords:** tixocortol, pivalone, crystal structure, polymorphs

## Abstract

Two polymorphs, (**I**) and (**II**), of (*S*)-{2-[(8*S*,9*S*,10*R*,11*S*,13*S*,14*S*,17*R*)-11,17-dihy­droxy-10,13-dimethyl-3-oxo-2,6,7,8,9,11,12,14,15,16-deca­hydro-1*H*-cyclo­penta­[*a*]phenanthren-17-yl]-2-oxoeth­yl} 2,2-di­methyl­propane­thio­ate, C_26_H_38_O_5_S, have been identified. They are ortho­rhom­bic, non-centrosymmetric (*P*2_1_2_1_2_1_). The structures display layers of mol­ecules conected *via* O—H⋯O hydrogen bonds along the *b-*axis direction in polymorph (**I**) and along the *c-*axis direction in polymorph (**II**). The structure of (**II**) exhibits disorder of the main mol­ecule.

## Abstract 2

Two ortho­rhom­bic polymorphs of the anti-inflammatory corticosteroid tixocortol pivalate have been identified. The two structures are characterized by layers of mol­ecules connected by strong O—H⋯O hydrogen bonds.

## Chemical context   

Tixocortol pivalate, also named Pivalone®, is a corticosteroid with local and topical anti-inflammatory activity (Davies *et al.*, 1981 ▸; Jezequel *et al.*, 1979 ▸; Liddle *et al.*, 1960 ▸; Maza­uric & Alligier, 1978 ▸; Nugent *et al.*, 1963 ▸; Uphill, 1981 ▸) equal to that of hydro­cortisone. As a corticosteroid, Tixocortol pivalate is used topically to relieve contact allergies and is also frequently recommended as a screening test for class A corticosteroids (Bircher *et al.*, 1995 ▸; Burden & Beck, 1992 ▸; Lauerma, 1991 ▸; Bouley, 2013 ▸). Surprisingly, the structure of tixocortol pivalate has never been determined. It was therefore of inter­est to obtain two polymorphs, (**I**) and (**II**), of the title compound prepared by total enantio-selective synthesis. 
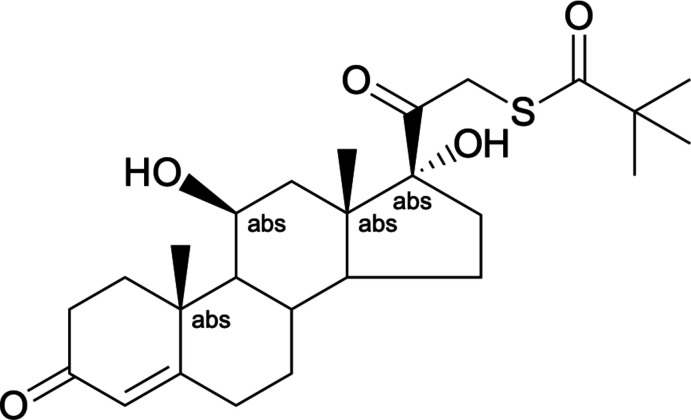


## Structural commentary   

**Figure 1:**
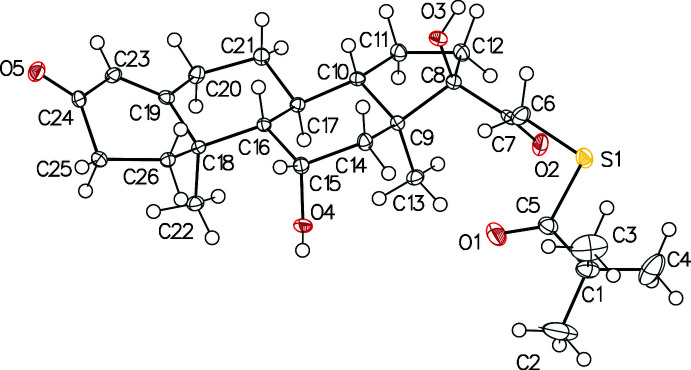
*ORTEP* view of polymorph (**I**). Displacement ellipsoids are drawn at the 50% probability level.

The presence of two polymorphs was confirmed by powder X-ray diffraction (PXRD) and the structures were determined by single crystal X-ray diffraction (SCXRD). The absolute configuration of its seven asymmetric carbons was established. Both polymorphs of the title compound consist of a (*S*)-{2-[(8*S*,9*S*,10*R*,11*S*,13*S*,14*S*,17*R*)-11,17-dihy­droxy-10,13-dimeth­yl-3-oxo-2,6,7,8,9,11,12,14,15,16-deca­hydro-1*H*-cyclo­penta­[*a*]phenanthren-17-yl]-2-oxoeth­yl} 2,2-di­methyl­propane­thio­ate mol­ecule in the asymmetric unit (Figs. 1 ▸ and 2 ▸). The general shape of the mol­ecule is strongly influenced by the conformation of one five-membered ring and three six-membered rings. In both polymorphs (Table 1 ▸), the five-membered ring (C8–C12) adopts an envelope form, both central six-membered rings (C9/C14–C17/C10 and C16/C18–C21/C17) adopt chair conformations and the six-membered ring with the double bond (C18/C19/C23–C26) adopts a half-chair conformation (Cremer & Pople, 1975 ▸). The superposition of the mol­ecules, with the Automatic Mol­ecule Overlay feature of *Mercury* (Macrae *et al.*, 2020 ▸), results in an r.m.s.d. of 0.829 and a maximum deviation of 2.545 Å if no flexibility is allowed and in values of 0.336 and 0.856, respectively, if flexibility is allowed. The main difference is on the dimethyl-sulfanyl-propanone group whose position is imposed by crystal packing.

## Supra­molecular features   

**Figure 3:**
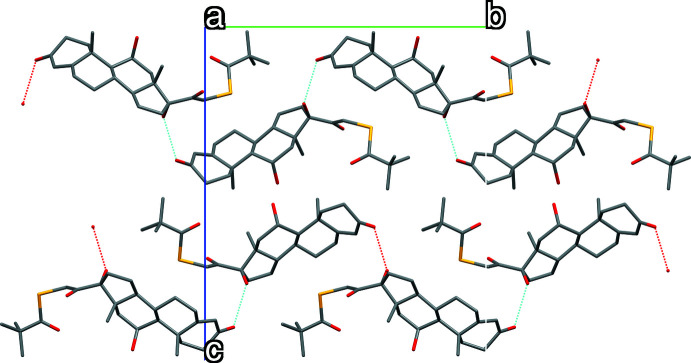
View of the hydrogen bond-network in polymorph (**I**).

The crystal packing in both structures is stabilized by one O—H⋯O hydrogen bond (Figs. 3 ▸ and 4 ▸, Tables 2 ▸ and 3 ▸) producing layers along (010) for polymorph (**I**) (PL358) and along (001) for polymorph (**II**) (SY20C174). The geometry of these inter­actions indicates that these are strong hydrogen bonds.

## Morphology prediction   

**Figure 5:**
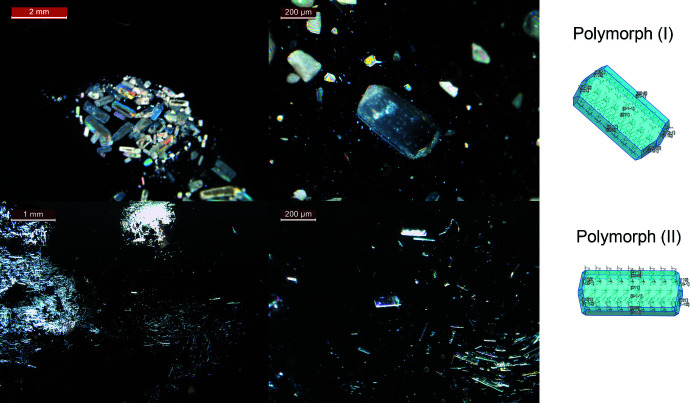
View of the crystal morphology of polymorph (**I**) (top) and (**II**) (bottom).

In both polymorphs, it was observed that the same type of hydrogen bonds plays a dominant role in the formation of hydrogen-bonded networks. However, the arrangements of mol­ecules in the crystal packing of polymorphs (**I**) and (**II**) are different. The different arrangements can also be seen in the external shape and size of the crystals. The theoretical crystal habits of polymorphs (**I**) and (**II**) were predicted based on the BFDH model with *Mercury* (Fig. 5 ▸). The morphologies of Pivalone polymorphs (**I**) and (**II**) display significant differences in their main crystal dimension.

## Synthesis and crystallization   

**Figure 6:**
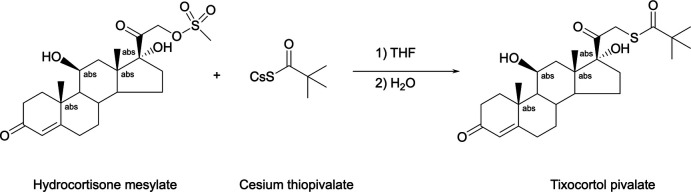
Reaction scheme for the synthesis of tixocortol pivalate.

Tixocortol pivalate (Fig. 6 ▸) has been produced as follows (Bouley, 2013 ▸): in a dry inerted flask, cesium thio­pivalate (620 g, 2.48 mol) and tetra­hydro­furan (1460 mL) are stirred at room temperature. A hydro­cortisone mesylate (995 g, 2.26 mol) solution in THF (4600 mL) is added in 1 h below 293 K. After 16 h of stirring, the reaction mixture is cooled below 283 K and water (12320 mL) is added. After addition, the reaction mixture is stirred for approximately 2 h. The precipitate is filtered and washed with water (10 × 820 mL). After drying under vacuum at 323 K for one night, the product is isolated as a white powder (yield 93%, purity by HPLC 98.5%).

## Powder X-ray diffraction (PXRD)   

Analyses were performed at room temperature from 2θ = 3 to 50° with an increasing step size of 0.02° and a count time of 120 s. The X-ray powder diffraction patterns were registered in transmission mode unless mentioned otherwise. The samples (few milligrams) are introduced without being crushed in 1 mm diameter glass capillaries to avoid preferential orientation. The capillaries are sealed to avoid contact with air. The analysis is performed in transmission mode by using a focusing X-ray mirror with divergence slits and anti-scatter slits (aperture 0.5°), on an Empyrean diffractometer from PANalytical Company (PANalytical, 2011 ▸) equipped with a copper anti­cathode tube (wavelength λ *K*α1 = 1.54060 Å/*K*α2 = 1.54443 Å) and with a PIXcel 1D detector with anti-scatter slits of 7.5 mm. The calibration of the analytical instrument is checked before each analytical batch according to quality systems.

**Figure 7:**
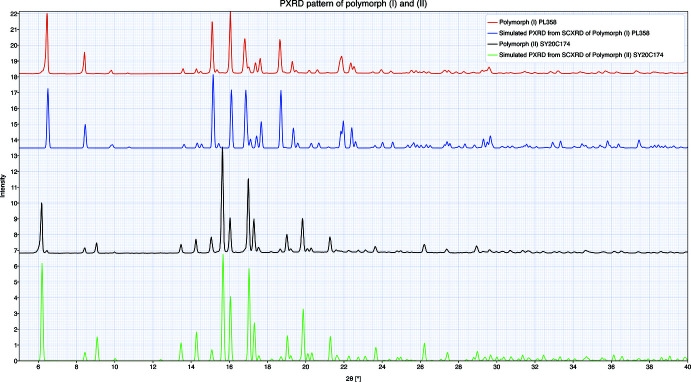
PXRD patterns of polymorphs (**I**) and (**II**) and their simulated patterns from the SCXRD study at room temperature.

Unit-cell parameters were obtained using indexing methods included in *ITO* (Visser, 1969 ▸) or *DICVOL* (Boultif & Louër, 2004 ▸). Le Bail (Le Bail, 1988 ▸) refinement was performed by using *JANA2006* (Petříček *et al.*, 2014 ▸) with the most plausible unit cell. The cell parameters found at room temperature were compared to those found from single crystal at different temperatures (Table 4 ▸). The cell parameters at low temperature and at ambient temperature found from single crystal and from powder diffraction are similar, confirming that no phase change occurs with different temperatures. The simulated PXRD patterns were calculated (Palmer, 2015 ▸) from SCXRD with cell parameters obtained at room temperature (Fig. 7 ▸).

## Structure solution and refinement   

Crystal data, data collection and structure refinement details are summarized in Table 5 ▸. The dimethyl-sulfanyl-propanone group was found to be disordered over two positions 77 (1)%/23 (1)% in polymotph (**II**). The SAME (Sheldrick, 2015*b* ▸) restraint was employed for the minor disordered part to maintain a reasonable model. All non-hydrogen atoms were refined anisotropically, except the minor disorder component. Hydrogen-atom positions were calculated geometrically and refined using the riding model. All H atoms, on carbon atoms, were placed at calculated positions using a riding model with C—H = 0.95 Å (aromatic), 0.99 Å (methyl­ene) or 1 Å (methine) with *U*_iso_(H) = 1.2*U*_eq_(C). H atoms on oxygen atoms were located in difference-Fourier maps. Their positional parameters were refined as an idealized OH group (AFIX 147), (Sheldrick, 2015*b* ▸) with *U*_iso_(H) = 1.5*U*_eq_(O). The TWIN/BASF instruction was used to refine the Flack parameter.

## Supplementary Material

Crystal structure: contains datablock(s) PL358, SY20C174, New_Global_Publ_Block. DOI: 10.1107/S2056989021007167/dj2019sup1.cif

Structure factors: contains datablock(s) PL358. DOI: 10.1107/S2056989021007167/dj2019PL358sup2.hkl

Supporting information file. DOI: 10.1107/S2056989021007167/dj2019PL358sup4.cdx

Structure factors: contains datablock(s) SY20C174. DOI: 10.1107/S2056989021007167/dj2019SY20C174sup3.hkl

Supporting information file. DOI: 10.1107/S2056989021007167/dj2019SY20C174sup5.cdx

CCDC references: 2095871, 2095870

Additional supporting information:  crystallographic information; 3D view; checkCIF report

## Figures and Tables

**Figure 2:**
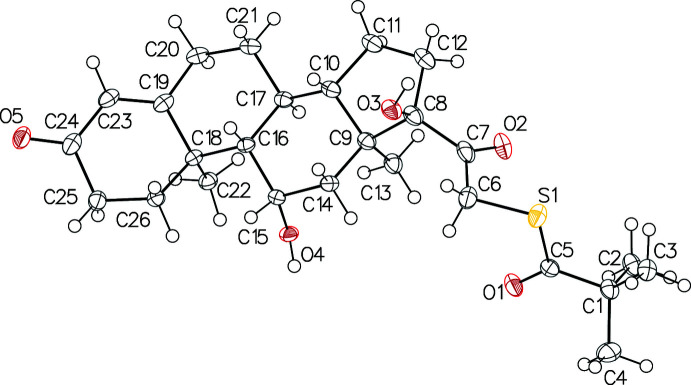
*ORTEP* view of polymorph (**II**). Displacement ellipsoids are drawn at the 30% probability level. The minor component of the disorder is omitted for clarity.

**Figure 4:**
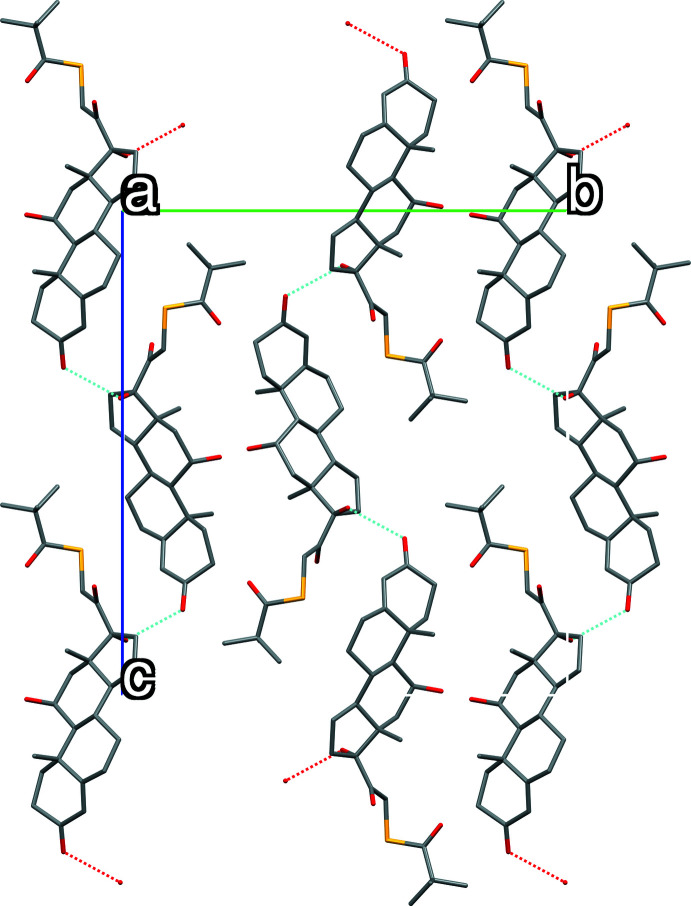
View of the hydrogen-bond network in polymorph (**II**).

**Table 1:** Ring puckering parameters

Compound	PL358 (**I**)	SY20C174 (**II**)
C8–C12	*Q*2 = 0.4847 (18) Å	*Q*2 = 0.441 (5) Å
Envelope conformation	φ2 = 39.4 (2)°	φ2 = 41.4 (6)°
C9/C14–C17/C10	*Q* = 0.5519 (17) Å | Θ = 9.45 (18) ° | φ2 = 53.2 (11)°	*Q* = 0.556 (4) Å | Θ = 13.4 (4) ° | φ2 = 37 (2)°
Chair conformation	*Q*2 = 0.0908 (17) Å | *Q*3 = 54.4444 (17) Å | φ2 = 53.2 (11)°	*Q*2 = 0.128 (4) Å | *Q*3 = 0.541 (4) Å | φ2 = 37 (2)°
C16/C18–C21/C17	*Q* = 0.5450 (17) Å | Θ = 175.11 (18) ° | φ2 = 170 (2)°	*Q* = 0.538 (4) Å | Θ = 173.2 (4) ° | φ2 = 196 (4)°
Chair conformation	*Q*2 = 0.0457 (17) Å | *Q*3 = −0.5431 (17) Å | φ2 = 170 (2)°	*Q*2 = 0.0065 (4) Å | *Q*3 = −0.534 (4) Å | φ2 = 196 (4)°
C18/C19/C23–C26	*Q* = 0.4724 (18) Å | Θ = 52.7 (2) ° | φ2 = 266.8 (3)°	*Q* = 0.454 (4) Å | Θ = 55.6 (5) ° | φ2 = 281.9 (7)°
Half-chair conformation	*Q*2 = 0.3756 (18) Å | *Q*3 = 0.2865 (18) Å | φ2 = 266.8 (3)°	*Q*2 = 0.375 (4) Å | *Q*3 = 0.256 (4) Å | φ2 = 281.9 (7)°

**Table 2:** Hydrogen-bond geometry (Å, °) for PL358 (**I**)

*D*—H⋯*A*	*D*—H	H⋯*A*	*D*⋯*A*	*D*—H⋯*A*
O3—H3⋯O5^i^	0.84	2.07	2.9021 (17)	169

Symmetry code: (i) -x+1, y+{\script{1\over 2}}, -z+{\script{1\over 2}}.

**Table 3:** Hydrogen-bond geometry (Å, °) for SY20C174 (**II**)

*D*—H⋯*A*	*D*—H	H⋯*A*	*D*⋯*A*	*D*—H⋯*A*
O3—H3⋯O5^i^	0.84	1.96	2.802 (4)	175

Symmetry code: (i) -x+{\script{1\over 2}}, -y+1, z+{\script{1\over 2}}.

**Table 4:** Cell parameters determined from SCXRD and PXRD at different temperatures

Compound	PL358	PL358	PL358	SY20C174	SY20C174	SY20C174
XRD measurement	SCXRD	SCXRD	PXRD	SCXRD	SCXRD	PXRD
Temperature	110 K	295 K	295 K	100 K	298 K	295 K
Space group	*P*2_1_2_1_2_1_	*P*2_1_2_1_2_1_	*P*2_1_2_1_2_1_	*P*2_1_2_1_2_1_	*P*2_1_2_1_2_1_	*P*2_1_2_1_2_1_
*a*	6.4201 (2)	6.467 (5)	6.4775 (2)	6.0146 (2)	6.157 (9)	6.1573 (2)
*b*	17.6239 (7)	17.887 (12)	17.9583 (7)	19.2817 (7)	19.46 (3)	19.4684 (7)
*c*	20.8997 (8)	20.897 (15)	20.9335 (7)	20.9887 (7)	20.92 (3)	20.8859 (9)
Volume	2364.7 (1)	2417 (5)	2435.1 (1)	2434.1 (1)	2508 (11)	2503.7 (2)

**Table 5:** Experimental details

	PL358 (**I**)	SY20C174 (**II**)
Crystal data		
Chemical formula	C_26_H_38_O_5_S	C_26_H_38_O_5_S
*M*_r_	462.62	462.62
Crystal system, space group	Orthorhombic, *P*2_1_2_1_2_1_	Orthorhombic, *P*2_1_2_1_2_1_
Temperature (K)	110	100
*a*, *b*, *c* (Å)	6.4201 (2), 17.6239 (7), 20.8997 (8)	6.0146 (2), 19.2817 (7), 20.9887 (7)
*V* (Å^3^)	2364.74 (15)	2434.10 (14)
*Z*	4	4
Radiation type	Mo *K*α	Cu *K*α
μ (mm^−1^)	0.17	1.46
Crystal size (mm)	0.46 × 0.25 × 0.24	0.18 × 0.06 × 0.05

Data collection		
Diffractometer	Nonius Kappa APEXII	Bruker D8 Venture
Absorption correction	Multi-scan (*SADABS*; Krause *et al.*, 2015 ▸)	Multi-scan (*SADABS*; Krause *et al.*, 2015 ▸)
*T*_min_, *T*_max_	0.912, 0.958	0.707, 0.862
No. of measured, independent and observed [*I* > 2σ(*I*)] reflections	74424, 5424, 5180	30900, 4303, 3803
*R*_int_	0.034	0.102

Refinement		
*R*[*F* ^2^ > 2σ(*F* ^2^)], *wR*(*F* ^2^), *S*	0.028, 0.072, 1.04	0.055, 0.130, 1.07
No. of reflections	5424	4303
No. of parameters	296	329
No. of restraints	0	16
H-atom treatment	H-atom parameters constrained	H-atom parameters constrained
Δρ_max_, Δρ_min_ (e Å^−3^)	0.26, −0.23	0.26, −0.39
Absolute structure	Flack *x* determined using 2176 quotients [(*I* ^+^)−(*I* ^−^)]/[(*I* ^+^)+(*I* ^−^)] (Parsons *et al.*, 2013 ▸)	Flack *x* obtained from refinement
Absolute structure parameter	0.027 (13)	0.11 (4)

Computer programs: *APEX3* and *SAINT* (Bruker, 2016 ▸), *SHELXT* (Sheldrick, 2015*a* ▸), *SHELXL* (Sheldrick, 2015*b* ▸) and *OLEX2* (Dolomanov *et al.*, 2009 ▸).

## supplementary crystallographic information

### (S)-{2-[(8S,9S,10R,11S,13S,14S,17R)-11,17-Dihydroxy-10,13-dimethyl-3-oxo-2,6,7,8,9,11,12,14,15,16-decahydro-1H-cyclopenta[a]phenanthren-17-yl]-2-oxoethyl} 2,2-dimethylpropanethioate (PL358) Crystal data

**Table:**

C_26_H_38_O_5_S	*D*_x_ = 1.299 Mg m^−^^3^
*M**_r_* = 462.62	Mo *K*α radiation, λ = 0.71073 Å
Orthorhombic, *P*2_1_2_1_2_1_	Cell parameters from 9539 reflections
*a* = 6.4201 (2) Å	θ = 2.3–27.4°
*b* = 17.6239 (7) Å	µ = 0.17 mm^−^^1^
*c* = 20.8997 (8) Å	*T* = 110 K
*V* = 2364.74 (15) Å^3^	Prism, clear light colourless
*Z* = 4	0.46 × 0.25 × 0.24 mm
*F*(000) = 1000	

### (S)-{2-[(8S,9S,10R,11S,13S,14S,17R)-11,17-Dihydroxy-10,13-dimethyl-3-oxo-2,6,7,8,9,11,12,14,15,16-decahydro-1H-cyclopenta[a]phenanthren-17-yl]-2-oxoethyl} 2,2-dimethylpropanethioate (PL358) Data collection

**Table:**

Nonius Kappa APEXII diffractometer	5424 independent reflections
Radiation source: X-ray tube, Siemens KFF Mo 2K-180	5180 reflections with *I* > 2σ(*I*)
Graphite monochromator	*R*_int_ = 0.034
Detector resolution: 512 x 512 pixels mm^-1^	θ_max_ = 27.5°, θ_min_ = 3.0°
φ and ω scans'	*h* = −8→8
Absorption correction: multi-scan (SADABS; Krause *et al.*, 2015)	*k* = −22→22
*T*_min_ = 0.912, *T*_max_ = 0.958	*l* = −27→27
74424 measured reflections	

### (S)-{2-[(8S,9S,10R,11S,13S,14S,17R)-11,17-Dihydroxy-10,13-dimethyl-3-oxo-2,6,7,8,9,11,12,14,15,16-decahydro-1H-cyclopenta[a]phenanthren-17-yl]-2-oxoethyl} 2,2-dimethylpropanethioate (PL358) Refinement

**Table:**

Refinement on *F*^2^	Hydrogen site location: inferred from neighbouring sites
Least-squares matrix: full	H-atom parameters constrained
*R*[*F*^2^ > 2σ(*F*^2^)] = 0.028	*w* = 1/[σ^2^(*F*_o_^2^) + (0.0409*P*)^2^ + 0.6195*P*] where *P* = (*F*_o_^2^ + 2*F*_c_^2^)/3
*wR*(*F*^2^) = 0.072	(Δ/σ)_max_ < 0.001
*S* = 1.04	Δρ_max_ = 0.26 e Å^−^^3^
5424 reflections	Δρ_min_ = −0.22 e Å^−^^3^
296 parameters	Absolute structure: Flack *x* determined using 2176 quotients [(*I*^+^)-(*I*^-^)]/[(*I*^+^)+(*I*^-^)] (Parsons *et al.*, 2013)
0 restraints	Absolute structure parameter: 0.027 (13)
Primary atom site location: dual	

### (S)-{2-[(8S,9S,10R,11S,13S,14S,17R)-11,17-Dihydroxy-10,13-dimethyl-3-oxo-2,6,7,8,9,11,12,14,15,16-decahydro-1H-cyclopenta[a]phenanthren-17-yl]-2-oxoethyl} 2,2-dimethylpropanethioate (PL358) Special details

**Table:**

Geometry. All esds (except the esd in the dihedral angle between two l.s. planes) are estimated using the full covariance matrix. The cell esds are taken into account individually in the estimation of esds in distances, angles and torsion angles; correlations between esds in cell parameters are only used when they are defined by crystal symmetry. An approximate (isotropic) treatment of cell esds is used for estimating esds involving l.s. planes.

### (S)-{2-[(8S,9S,10R,11S,13S,14S,17R)-11,17-Dihydroxy-10,13-dimethyl-3-oxo-2,6,7,8,9,11,12,14,15,16-decahydro-1H-cyclopenta[a]phenanthren-17-yl]-2-oxoethyl} 2,2-dimethylpropanethioate (PL358) Fractional atomic coordinates and isotropic or equivalent isotropic displacement parameters (Å^2^)

**Table:**

	*x*	*y*	*z*	*U*_iso_*/*U*_eq_	
S1	0.57981 (8)	0.59132 (2)	0.30170 (2)	0.02260 (11)	
O3	0.6410 (2)	0.35175 (7)	0.23101 (6)	0.0154 (2)	
H3	0.671037	0.360570	0.192609	0.023*	
O5	0.2936 (2)	−0.10124 (7)	0.40054 (6)	0.0186 (3)	
O2	0.9568 (2)	0.49062 (7)	0.30543 (6)	0.0219 (3)	
O4	0.6656 (3)	0.25785 (7)	0.46752 (6)	0.0243 (3)	
H4	0.576170	0.276912	0.492065	0.036*	
O1	0.4852 (3)	0.52261 (8)	0.40864 (7)	0.0352 (4)	
C10	0.8689 (2)	0.24334 (9)	0.30097 (7)	0.0105 (3)	
H10	0.757227	0.229873	0.269614	0.013*	
C16	0.6649 (2)	0.16686 (9)	0.37858 (7)	0.0101 (3)	
H16	0.572073	0.153973	0.341722	0.012*	
C24	0.4122 (3)	−0.04919 (9)	0.41447 (8)	0.0133 (3)	
C22	0.7966 (3)	0.09961 (10)	0.48265 (8)	0.0139 (3)	
H22A	0.939136	0.110305	0.468479	0.021*	
H22B	0.793474	0.050904	0.505250	0.021*	
H22C	0.749690	0.139990	0.511490	0.021*	
C7	0.8049 (3)	0.45608 (10)	0.28770 (8)	0.0152 (3)	
C18	0.6505 (2)	0.09589 (9)	0.42376 (7)	0.0101 (3)	
C17	0.8818 (2)	0.17768 (9)	0.34806 (7)	0.0108 (3)	
H17	0.986748	0.189289	0.382019	0.013*	
C23	0.6075 (3)	−0.03662 (9)	0.37971 (8)	0.0133 (3)	
H23	0.657156	−0.075699	0.352367	0.016*	
C26	0.4233 (3)	0.08633 (9)	0.44563 (7)	0.0116 (3)	
H26A	0.395372	0.122300	0.480995	0.014*	
H26B	0.329792	0.099703	0.409691	0.014*	
C21	0.9454 (3)	0.10574 (9)	0.31190 (8)	0.0140 (3)	
H21A	0.855784	0.100246	0.273627	0.017*	
H21B	1.091004	0.111287	0.297053	0.017*	
C20	0.9280 (3)	0.03384 (9)	0.35248 (8)	0.0138 (3)	
H20A	0.950163	−0.011075	0.324867	0.017*	
H20B	1.038671	0.034177	0.385458	0.017*	
C9	0.8031 (3)	0.31896 (9)	0.33168 (7)	0.0110 (3)	
C11	1.0623 (3)	0.26354 (9)	0.26139 (8)	0.0142 (3)	
H11A	1.076384	0.229298	0.224088	0.017*	
H11B	1.190147	0.260105	0.287716	0.017*	
C1	0.4236 (3)	0.65833 (11)	0.41260 (8)	0.0219 (4)	
C19	0.7196 (2)	0.02742 (9)	0.38462 (8)	0.0112 (3)	
C15	0.5701 (3)	0.24038 (9)	0.40657 (8)	0.0142 (3)	
H15	0.418893	0.230653	0.414522	0.017*	
C5	0.4924 (3)	0.58328 (10)	0.38238 (9)	0.0184 (3)	
C14	0.5861 (3)	0.30893 (9)	0.36064 (8)	0.0134 (3)	
H14A	0.483971	0.302354	0.325584	0.016*	
H14B	0.548391	0.355646	0.384264	0.016*	
C25	0.3715 (3)	0.00567 (9)	0.46830 (8)	0.0138 (3)	
H25A	0.458872	−0.007601	0.505705	0.017*	
H25B	0.223523	0.002816	0.481369	0.017*	
C8	0.8139 (3)	0.37093 (9)	0.27071 (8)	0.0126 (3)	
C13	0.9608 (3)	0.34721 (9)	0.38172 (8)	0.0155 (3)	
H13A	0.975665	0.309141	0.415590	0.023*	
H13B	0.911232	0.394965	0.400299	0.023*	
H13C	1.096090	0.355534	0.361145	0.023*	
C12	1.0227 (3)	0.34651 (10)	0.23951 (8)	0.0155 (3)	
H12A	1.137374	0.379980	0.253852	0.019*	
H12B	1.012823	0.349257	0.192295	0.019*	
C6	0.5917 (3)	0.49264 (10)	0.28061 (9)	0.0205 (4)	
H6A	0.491554	0.464500	0.307701	0.025*	
H6B	0.546071	0.487033	0.235619	0.025*	
C4	0.5645 (4)	0.72401 (12)	0.39145 (14)	0.0431 (6)	
H4A	0.708534	0.713275	0.404017	0.065*	
H4B	0.518021	0.771070	0.411956	0.065*	
H4C	0.556793	0.729660	0.344859	0.065*	
C3	0.2026 (4)	0.67460 (16)	0.38883 (13)	0.0430 (6)	
H3A	0.203839	0.679901	0.342158	0.065*	
H3B	0.151797	0.721734	0.408214	0.065*	
H3C	0.110650	0.632594	0.400915	0.065*	
C2	0.4239 (6)	0.64969 (16)	0.48501 (10)	0.0564 (9)	
H2A	0.332983	0.607392	0.497155	0.085*	
H2B	0.372756	0.696584	0.504713	0.085*	
H2C	0.565977	0.639511	0.499814	0.085*	

### (S)-{2-[(8S,9S,10R,11S,13S,14S,17R)-11,17-Dihydroxy-10,13-dimethyl-3-oxo-2,6,7,8,9,11,12,14,15,16-decahydro-1H-cyclopenta[a]phenanthren-17-yl]-2-oxoethyl} 2,2-dimethylpropanethioate (PL358) Atomic displacement parameters (Å^2^)

**Table:**

	*U*^11^	*U*^22^	*U*^33^	*U*^12^	*U*^13^	*U*^23^
S1	0.0352 (3)	0.01312 (19)	0.0195 (2)	0.00396 (19)	0.01024 (19)	0.00171 (16)
O3	0.0179 (6)	0.0176 (6)	0.0106 (5)	−0.0006 (5)	−0.0030 (5)	0.0007 (4)
O5	0.0195 (6)	0.0148 (6)	0.0216 (6)	−0.0049 (5)	0.0009 (5)	−0.0018 (5)
O2	0.0258 (7)	0.0141 (6)	0.0257 (7)	−0.0061 (5)	−0.0009 (6)	0.0018 (5)
O4	0.0479 (9)	0.0151 (6)	0.0100 (6)	0.0015 (6)	0.0044 (6)	−0.0025 (5)
O1	0.0617 (11)	0.0218 (7)	0.0221 (7)	0.0018 (7)	0.0063 (7)	0.0068 (6)
C10	0.0096 (7)	0.0121 (7)	0.0099 (7)	0.0001 (6)	0.0002 (6)	−0.0007 (6)
C16	0.0111 (7)	0.0097 (7)	0.0093 (6)	0.0006 (6)	0.0011 (6)	−0.0002 (6)
C24	0.0172 (8)	0.0099 (7)	0.0128 (7)	0.0012 (6)	−0.0020 (7)	0.0024 (6)
C22	0.0157 (8)	0.0138 (8)	0.0122 (7)	0.0002 (6)	−0.0027 (6)	−0.0015 (6)
C7	0.0225 (9)	0.0147 (8)	0.0082 (7)	0.0000 (7)	0.0026 (7)	0.0028 (6)
C18	0.0114 (7)	0.0095 (7)	0.0094 (7)	−0.0004 (6)	−0.0006 (5)	−0.0010 (6)
C17	0.0107 (7)	0.0106 (7)	0.0111 (7)	0.0006 (6)	0.0007 (6)	−0.0006 (6)
C23	0.0156 (8)	0.0113 (7)	0.0132 (7)	0.0012 (6)	0.0003 (6)	−0.0023 (6)
C26	0.0128 (7)	0.0108 (7)	0.0111 (7)	−0.0001 (6)	0.0012 (6)	−0.0009 (6)
C21	0.0142 (7)	0.0125 (7)	0.0154 (7)	0.0012 (6)	0.0045 (6)	−0.0003 (6)
C20	0.0127 (7)	0.0111 (7)	0.0177 (8)	0.0018 (6)	0.0032 (7)	−0.0018 (6)
C9	0.0125 (7)	0.0104 (7)	0.0102 (7)	−0.0005 (6)	−0.0004 (6)	0.0007 (6)
C11	0.0128 (7)	0.0149 (7)	0.0149 (7)	−0.0006 (7)	0.0030 (6)	0.0006 (6)
C1	0.0255 (9)	0.0243 (9)	0.0158 (8)	0.0083 (8)	−0.0004 (8)	−0.0027 (7)
C19	0.0124 (7)	0.0119 (7)	0.0092 (7)	0.0028 (6)	−0.0014 (6)	0.0004 (6)
C15	0.0167 (8)	0.0116 (7)	0.0142 (7)	0.0013 (6)	0.0045 (7)	−0.0001 (6)
C5	0.0209 (8)	0.0195 (8)	0.0146 (7)	0.0021 (7)	−0.0015 (7)	0.0016 (7)
C14	0.0141 (7)	0.0111 (7)	0.0151 (7)	0.0019 (6)	0.0024 (6)	0.0010 (6)
C25	0.0154 (8)	0.0140 (8)	0.0119 (7)	−0.0013 (6)	0.0028 (6)	0.0000 (6)
C8	0.0140 (8)	0.0126 (7)	0.0110 (7)	−0.0012 (6)	−0.0016 (6)	0.0007 (6)
C13	0.0195 (8)	0.0138 (7)	0.0133 (7)	−0.0007 (7)	−0.0046 (7)	−0.0013 (6)
C12	0.0170 (8)	0.0150 (8)	0.0143 (8)	−0.0013 (7)	0.0035 (6)	0.0012 (6)
C6	0.0276 (9)	0.0137 (8)	0.0203 (8)	0.0030 (7)	−0.0007 (8)	−0.0039 (6)
C4	0.0458 (14)	0.0216 (10)	0.0621 (16)	0.0019 (10)	0.0085 (14)	−0.0157 (10)
C3	0.0298 (12)	0.0568 (16)	0.0424 (13)	0.0196 (11)	−0.0046 (11)	−0.0140 (12)
C2	0.105 (3)	0.0496 (15)	0.0150 (9)	0.0370 (18)	−0.0069 (13)	−0.0052 (9)

### (S)-{2-[(8S,9S,10R,11S,13S,14S,17R)-11,17-Dihydroxy-10,13-dimethyl-3-oxo-2,6,7,8,9,11,12,14,15,16-decahydro-1H-cyclopenta[a]phenanthren-17-yl]-2-oxoethyl} 2,2-dimethylpropanethioate (PL358) Geometric parameters (Å, º)

**Table:**

S1—C5	1.7827 (18)	C20—H20A	0.9900
S1—C6	1.7957 (18)	C20—H20B	0.9900
O3—H3	0.8400	C20—C19	1.501 (2)
O3—C8	1.426 (2)	C9—C14	1.529 (2)
O5—C24	1.227 (2)	C9—C8	1.571 (2)
O2—C7	1.208 (2)	C9—C13	1.538 (2)
O4—H4	0.8400	C11—H11A	0.9900
O4—C15	1.447 (2)	C11—H11B	0.9900
O1—C5	1.203 (2)	C11—C12	1.553 (2)
C10—H10	1.0000	C1—C5	1.531 (3)
C10—C17	1.521 (2)	C1—C4	1.534 (3)
C10—C9	1.538 (2)	C1—C3	1.531 (3)
C10—C11	1.534 (2)	C1—C2	1.521 (3)
C16—H16	1.0000	C15—H15	1.0000
C16—C18	1.570 (2)	C15—C14	1.546 (2)
C16—C17	1.543 (2)	C14—H14A	0.9900
C16—C15	1.546 (2)	C14—H14B	0.9900
C24—C23	1.466 (2)	C25—H25A	0.9900
C24—C25	1.506 (2)	C25—H25B	0.9900
C22—H22A	0.9800	C8—C12	1.552 (2)
C22—H22B	0.9800	C13—H13A	0.9800
C22—H22C	0.9800	C13—H13B	0.9800
C22—C18	1.549 (2)	C13—H13C	0.9800
C7—C8	1.543 (2)	C12—H12A	0.9900
C7—C6	1.520 (3)	C12—H12B	0.9900
C18—C26	1.538 (2)	C6—H6A	0.9900
C18—C19	1.524 (2)	C6—H6B	0.9900
C17—H17	1.0000	C4—H4A	0.9800
C17—C21	1.531 (2)	C4—H4B	0.9800
C23—H23	0.9500	C4—H4C	0.9800
C23—C19	1.342 (2)	C3—H3A	0.9800
C26—H26A	0.9900	C3—H3B	0.9800
C26—H26B	0.9900	C3—H3C	0.9800
C26—C25	1.535 (2)	C2—H2A	0.9800
C21—H21A	0.9900	C2—H2B	0.9800
C21—H21B	0.9900	C2—H2C	0.9800
C21—C20	1.529 (2)		
			
C5—S1—C6	99.71 (9)	C5—C1—C4	111.30 (16)
C8—O3—H3	109.5	C3—C1—C5	107.18 (17)
C15—O4—H4	109.5	C3—C1—C4	108.15 (19)
C17—C10—H10	106.4	C2—C1—C5	108.89 (16)
C17—C10—C9	113.83 (13)	C2—C1—C4	111.2 (2)
C17—C10—C11	118.79 (13)	C2—C1—C3	110.0 (2)
C9—C10—H10	106.4	C23—C19—C18	123.42 (15)
C11—C10—H10	106.4	C23—C19—C20	120.46 (15)
C11—C10—C9	104.26 (13)	C20—C19—C18	116.09 (13)
C18—C16—H16	104.3	O4—C15—C16	110.17 (13)
C17—C16—H16	104.3	O4—C15—H15	107.5
C17—C16—C18	113.58 (13)	O4—C15—C14	110.62 (13)
C17—C16—C15	114.07 (13)	C16—C15—H15	107.5
C15—C16—H16	104.3	C16—C15—C14	113.19 (13)
C15—C16—C18	114.64 (12)	C14—C15—H15	107.5
O5—C24—C23	121.75 (15)	O1—C5—S1	120.96 (15)
O5—C24—C25	123.32 (16)	O1—C5—C1	124.65 (17)
C23—C24—C25	114.93 (14)	C1—C5—S1	114.36 (13)
H22A—C22—H22B	109.5	C9—C14—C15	113.34 (13)
H22A—C22—H22C	109.5	C9—C14—H14A	108.9
H22B—C22—H22C	109.5	C9—C14—H14B	108.9
C18—C22—H22A	109.5	C15—C14—H14A	108.9
C18—C22—H22B	109.5	C15—C14—H14B	108.9
C18—C22—H22C	109.5	H14A—C14—H14B	107.7
O2—C7—C8	122.04 (17)	C24—C25—C26	109.05 (13)
O2—C7—C6	122.91 (16)	C24—C25—H25A	109.9
C6—C7—C8	115.05 (15)	C24—C25—H25B	109.9
C22—C18—C16	114.12 (13)	C26—C25—H25A	109.9
C26—C18—C16	108.77 (12)	C26—C25—H25B	109.9
C26—C18—C22	110.05 (12)	H25A—C25—H25B	108.3
C19—C18—C16	106.91 (12)	O3—C8—C7	109.59 (14)
C19—C18—C22	106.50 (13)	O3—C8—C9	107.43 (13)
C19—C18—C26	110.42 (13)	O3—C8—C12	111.23 (13)
C10—C17—C16	108.20 (12)	C7—C8—C9	112.25 (13)
C10—C17—H17	109.9	C7—C8—C12	113.50 (14)
C10—C17—C21	108.97 (13)	C12—C8—C9	102.54 (13)
C16—C17—H17	109.9	C9—C13—H13A	109.5
C21—C17—C16	110.02 (13)	C9—C13—H13B	109.5
C21—C17—H17	109.9	C9—C13—H13C	109.5
C24—C23—H23	118.4	H13A—C13—H13B	109.5
C19—C23—C24	123.21 (15)	H13A—C13—H13C	109.5
C19—C23—H23	118.4	H13B—C13—H13C	109.5
C18—C26—H26A	108.9	C11—C12—H12A	110.5
C18—C26—H26B	108.9	C11—C12—H12B	110.5
H26A—C26—H26B	107.7	C8—C12—C11	106.20 (13)
C25—C26—C18	113.48 (13)	C8—C12—H12A	110.5
C25—C26—H26A	108.9	C8—C12—H12B	110.5
C25—C26—H26B	108.9	H12A—C12—H12B	108.7
C17—C21—H21A	109.0	S1—C6—H6A	108.5
C17—C21—H21B	109.0	S1—C6—H6B	108.5
H21A—C21—H21B	107.8	C7—C6—S1	115.15 (13)
C20—C21—C17	113.14 (13)	C7—C6—H6A	108.5
C20—C21—H21A	109.0	C7—C6—H6B	108.5
C20—C21—H21B	109.0	H6A—C6—H6B	107.5
C21—C20—H20A	109.2	C1—C4—H4A	109.5
C21—C20—H20B	109.2	C1—C4—H4B	109.5
H20A—C20—H20B	107.9	C1—C4—H4C	109.5
C19—C20—C21	112.07 (14)	H4A—C4—H4B	109.5
C19—C20—H20A	109.2	H4A—C4—H4C	109.5
C19—C20—H20B	109.2	H4B—C4—H4C	109.5
C10—C9—C8	98.89 (12)	C1—C3—H3A	109.5
C10—C9—C13	112.53 (13)	C1—C3—H3B	109.5
C14—C9—C10	108.37 (13)	C1—C3—H3C	109.5
C14—C9—C8	115.37 (13)	H3A—C3—H3B	109.5
C14—C9—C13	111.59 (13)	H3A—C3—H3C	109.5
C13—C9—C8	109.50 (13)	H3B—C3—H3C	109.5
C10—C11—H11A	110.9	C1—C2—H2A	109.5
C10—C11—H11B	110.9	C1—C2—H2B	109.5
C10—C11—C12	104.17 (13)	C1—C2—H2C	109.5
H11A—C11—H11B	108.9	H2A—C2—H2B	109.5
C12—C11—H11A	110.9	H2A—C2—H2C	109.5
C12—C11—H11B	110.9	H2B—C2—H2C	109.5
			
O3—C8—C12—C11	−88.38 (16)	C26—C18—C19—C23	−10.2 (2)
O5—C24—C23—C19	166.22 (16)	C26—C18—C19—C20	171.80 (13)
O5—C24—C25—C26	−136.63 (16)	C21—C20—C19—C18	−52.86 (18)
O2—C7—C8—O3	−159.55 (15)	C21—C20—C19—C23	129.03 (16)
O2—C7—C8—C9	81.2 (2)	C9—C10—C17—C16	59.62 (17)
O2—C7—C8—C12	−34.5 (2)	C9—C10—C17—C21	179.23 (13)
O2—C7—C6—S1	−0.9 (2)	C9—C10—C11—C12	−31.87 (16)
O4—C15—C14—C9	76.44 (17)	C9—C8—C12—C11	26.18 (16)
C10—C17—C21—C20	−170.47 (13)	C11—C10—C17—C16	−177.02 (13)
C10—C9—C14—C15	52.98 (17)	C11—C10—C17—C21	−57.42 (18)
C10—C9—C8—O3	72.69 (15)	C11—C10—C9—C14	168.13 (13)
C10—C9—C8—C7	−166.78 (14)	C11—C10—C9—C8	47.53 (15)
C10—C9—C8—C12	−44.62 (14)	C11—C10—C9—C13	−67.98 (16)
C10—C11—C12—C8	2.91 (17)	C19—C18—C26—C25	41.50 (17)
C16—C18—C26—C25	158.52 (13)	C15—C16—C18—C22	−71.13 (17)
C16—C18—C19—C23	−128.32 (16)	C15—C16—C18—C26	52.16 (17)
C16—C18—C19—C20	53.64 (17)	C15—C16—C18—C19	171.40 (13)
C16—C17—C21—C20	−51.99 (18)	C15—C16—C17—C10	−51.38 (17)
C16—C15—C14—C9	−47.76 (19)	C15—C16—C17—C21	−170.32 (13)
C24—C23—C19—C18	−3.6 (3)	C5—S1—C6—C7	−95.53 (14)
C24—C23—C19—C20	174.33 (15)	C14—C9—C8—O3	−42.61 (18)
C22—C18—C26—C25	−75.79 (17)	C14—C9—C8—C7	77.93 (18)
C22—C18—C19—C23	109.31 (17)	C14—C9—C8—C12	−159.91 (14)
C22—C18—C19—C20	−68.74 (17)	C25—C24—C23—C19	−14.5 (2)
C7—C8—C12—C11	147.50 (14)	C8—C7—C6—S1	178.95 (12)
C18—C16—C17—C10	174.74 (12)	C8—C9—C14—C15	162.72 (13)
C18—C16—C17—C21	55.80 (16)	C13—C9—C14—C15	−71.47 (17)
C18—C16—C15—O4	55.85 (18)	C13—C9—C8—O3	−169.48 (13)
C18—C16—C15—C14	−179.71 (13)	C13—C9—C8—C7	−48.95 (18)
C18—C26—C25—C24	−59.02 (17)	C13—C9—C8—C12	73.22 (15)
C17—C10—C9—C14	−60.92 (16)	C6—S1—C5—O1	9.35 (19)
C17—C10—C9—C8	178.48 (13)	C6—S1—C5—C1	−168.80 (14)
C17—C10—C9—C13	62.97 (17)	C6—C7—C8—O3	20.62 (19)
C17—C10—C11—C12	−159.84 (14)	C6—C7—C8—C9	−98.66 (17)
C17—C16—C18—C22	62.48 (17)	C6—C7—C8—C12	145.63 (15)
C17—C16—C18—C26	−174.24 (12)	C4—C1—C5—S1	−38.1 (2)
C17—C16—C18—C19	−55.00 (16)	C4—C1—C5—O1	143.8 (2)
C17—C16—C15—O4	−77.53 (16)	C3—C1—C5—S1	79.96 (19)
C17—C16—C15—C14	46.91 (19)	C3—C1—C5—O1	−98.1 (2)
C17—C21—C20—C19	50.36 (19)	C2—C1—C5—S1	−161.1 (2)
C23—C24—C25—C26	44.08 (19)	C2—C1—C5—O1	20.9 (3)

### (S)-{2-[(8S,9S,10R,11S,13S,14S,17R)-11,17-Dihydroxy-10,13-dimethyl-3-oxo-2,6,7,8,9,11,12,14,15,16-decahydro-1H-cyclopenta[a]phenanthren-17-yl]-2-oxoethyl} 2,2-dimethylpropanethioate (PL358) Hydrogen-bond geometry (Å, º)

**Table:**

*D*—H···*A*	*D*—H	H···*A*	*D*···*A*	*D*—H···*A*
O3—H3···O5^i^	0.84	2.07	2.9021 (17)	169

Symmetry code: (i) −*x*+1, *y*+1/2, −*z*+1/2.

### (S)-{2-[(8S,9S,10R,11S,13S,14S,17R)-11,17-Dihydroxy-10,13-dimethyl-3-oxo-2,6,7,8,9,11,12,14,15,16-decahydro-1H-cyclopenta[a]phenanthren-17-yl]-2-oxoethyl} 2,2-dimethylpropanethioate (SY20C174) Crystal data

**Table:**

C_26_H_38_O_5_S	*D*_x_ = 1.262 Mg m^−^^3^
*M**_r_* = 462.62	Cu *K*α radiation, λ = 1.54178 Å
Orthorhombic, *P*2_1_2_1_2_1_	Cell parameters from 6178 reflections
*a* = 6.0146 (2) Å	θ = 3.1–66.5°
*b* = 19.2817 (7) Å	µ = 1.46 mm^−^^1^
*c* = 20.9887 (7) Å	*T* = 100 K
*V* = 2434.10 (14) Å^3^	Plate, clear light colourless
*Z* = 4	0.18 × 0.06 × 0.05 mm
*F*(000) = 1000	

### (S)-{2-[(8S,9S,10R,11S,13S,14S,17R)-11,17-Dihydroxy-10,13-dimethyl-3-oxo-2,6,7,8,9,11,12,14,15,16-decahydro-1H-cyclopenta[a]phenanthren-17-yl]-2-oxoethyl} 2,2-dimethylpropanethioate (SY20C174) Data collection

**Table:**

Bruker D8 Venture diffractometer	4303 independent reflections
Radiation source: sealed X-ray tube, high brilliance microfocus sealed tube, Cu	3803 reflections with *I* > 2σ(*I*)
QUAZAR MX multilayer optics monochromator	*R*_int_ = 0.102
Detector resolution: 1024 x 1024 pixels mm^-1^	θ_max_ = 66.7°, θ_min_ = 3.1°
φ and ω scans'	*h* = −7→6
Absorption correction: multi-scan (SADABS; Krause *et al.*, 2015)	*k* = −22→22
*T*_min_ = 0.707, *T*_max_ = 0.862	*l* = −24→25
30900 measured reflections	

### (S)-{2-[(8S,9S,10R,11S,13S,14S,17R)-11,17-Dihydroxy-10,13-dimethyl-3-oxo-2,6,7,8,9,11,12,14,15,16-decahydro-1H-cyclopenta[a]phenanthren-17-yl]-2-oxoethyl} 2,2-dimethylpropanethioate (SY20C174) Refinement

**Table:**

Refinement on *F*^2^	Hydrogen site location: inferred from neighbouring sites
Least-squares matrix: full	H-atom parameters constrained
*R*[*F*^2^ > 2σ(*F*^2^)] = 0.055	*w* = 1/[σ^2^(*F*_o_^2^) + (0.052*P*)^2^ + 1.3292*P*] where *P* = (*F*_o_^2^ + 2*F*_c_^2^)/3
*wR*(*F*^2^) = 0.130	(Δ/σ)_max_ < 0.001
*S* = 1.07	Δρ_max_ = 0.26 e Å^−^^3^
4303 reflections	Δρ_min_ = −0.39 e Å^−^^3^
329 parameters	Absolute structure: Flack *x* obtained from refinement
16 restraints	Absolute structure parameter: 0.11 (4)
Primary atom site location: dual	

### (S)-{2-[(8S,9S,10R,11S,13S,14S,17R)-11,17-Dihydroxy-10,13-dimethyl-3-oxo-2,6,7,8,9,11,12,14,15,16-decahydro-1H-cyclopenta[a]phenanthren-17-yl]-2-oxoethyl} 2,2-dimethylpropanethioate (SY20C174) Special details

**Table:**

Geometry. All esds (except the esd in the dihedral angle between two l.s. planes) are estimated using the full covariance matrix. The cell esds are taken into account individually in the estimation of esds in distances, angles and torsion angles; correlations between esds in cell parameters are only used when they are defined by crystal symmetry. An approximate (isotropic) treatment of cell esds is used for estimating esds involving l.s. planes.
Refinement. Refined as a 2-component inversion twin.

### (S)-{2-[(8S,9S,10R,11S,13S,14S,17R)-11,17-Dihydroxy-10,13-dimethyl-3-oxo-2,6,7,8,9,11,12,14,15,16-decahydro-1H-cyclopenta[a]phenanthren-17-yl]-2-oxoethyl} 2,2-dimethylpropanethioate (SY20C174) Fractional atomic coordinates and isotropic or equivalent isotropic displacement parameters (Å^2^)

**Table:**

	*x*	*y*	*z*	*U*_iso_*/*U*_eq_	Occ. (<1)
O2	0.5899 (6)	0.4360 (2)	0.72379 (16)	0.0542 (10)	
O3	0.2156 (5)	0.50617 (16)	0.61339 (15)	0.0390 (7)	
H3	0.209393	0.545661	0.630189	0.059*	
O4	0.4523 (5)	0.28363 (14)	0.49153 (14)	0.0367 (7)	
H4	0.369010	0.253012	0.507049	0.055*	
O5	0.2955 (7)	0.36601 (17)	0.17678 (14)	0.0487 (9)	
C19	0.5824 (7)	0.4099 (2)	0.3207 (2)	0.0332 (9)	
C23	0.5075 (8)	0.4126 (2)	0.2605 (2)	0.0384 (10)	
H23	0.550048	0.451088	0.235126	0.046*	
C10	0.5508 (7)	0.4744 (2)	0.5179 (2)	0.0324 (9)	
H10	0.415291	0.501122	0.505311	0.039*	
C26	0.2974 (7)	0.3182 (2)	0.34159 (18)	0.0312 (9)	
H26A	0.270014	0.275824	0.366990	0.037*	
H26B	0.167597	0.349225	0.347184	0.037*	
C21	0.7010 (7)	0.4915 (2)	0.4084 (2)	0.0346 (9)	
H21A	0.832545	0.517167	0.424124	0.042*	
H21B	0.578497	0.525229	0.402795	0.042*	
C18	0.5052 (7)	0.3548 (2)	0.36772 (19)	0.0288 (9)	
C24	0.3664 (8)	0.3607 (2)	0.2319 (2)	0.0406 (11)	
C15	0.3269 (7)	0.34602 (19)	0.48133 (19)	0.0296 (9)	
H15	0.180184	0.332125	0.462922	0.036*	
C8	0.4270 (8)	0.4760 (2)	0.6258 (2)	0.0386 (10)	
C17	0.6327 (7)	0.4380 (2)	0.4582 (2)	0.0292 (9)	
H17	0.763081	0.408045	0.468950	0.035*	
C9	0.4772 (7)	0.4237 (2)	0.5707 (2)	0.0322 (10)	
C22	0.6971 (8)	0.3019 (2)	0.3744 (2)	0.0345 (9)	
H22A	0.751711	0.289182	0.331957	0.052*	
H22B	0.642942	0.260357	0.396306	0.052*	
H22C	0.818350	0.322694	0.399048	0.052*	
C11	0.7052 (8)	0.5257 (2)	0.5512 (2)	0.0387 (10)	
H11A	0.698529	0.571722	0.530401	0.046*	
H11B	0.860739	0.508887	0.550330	0.046*	
C20	0.7564 (7)	0.4589 (2)	0.3438 (2)	0.0378 (11)	
H20A	0.775716	0.496321	0.311979	0.045*	
H20B	0.899444	0.433782	0.347301	0.045*	
C12	0.6174 (8)	0.5295 (3)	0.6202 (2)	0.0448 (12)	
H12A	0.561469	0.576745	0.629549	0.054*	
H12B	0.737654	0.518446	0.650724	0.054*	
C6	0.2076 (9)	0.4088 (2)	0.7138 (2)	0.0441 (11)	
H6AA	0.196918	0.361138	0.696528	0.053*	0.770 (4)
H6AB	0.082226	0.435978	0.696182	0.053*	0.770 (4)
H6BC	0.120508	0.394023	0.676151	0.053*	0.230 (4)
H6BD	0.119404	0.443918	0.737153	0.053*	0.230 (4)
C13	0.6669 (8)	0.3752 (2)	0.5918 (2)	0.0381 (10)	
H13A	0.721108	0.348936	0.554993	0.057*	
H13B	0.611557	0.343021	0.624265	0.057*	
H13C	0.788640	0.402879	0.609613	0.057*	
C25	0.3151 (8)	0.2983 (2)	0.27161 (19)	0.0373 (10)	
H25A	0.433983	0.263261	0.266140	0.045*	
H25B	0.173190	0.277449	0.257299	0.045*	
C14	0.2796 (7)	0.3837 (2)	0.54478 (19)	0.0303 (9)	
H14A	0.154299	0.416247	0.538403	0.036*	
H14B	0.232769	0.349021	0.576884	0.036*	
C7	0.4242 (9)	0.4410 (2)	0.6919 (2)	0.0421 (11)	
C16	0.4439 (7)	0.39282 (19)	0.43159 (19)	0.0270 (9)	
H16	0.326901	0.426958	0.418849	0.032*	
S1	0.1815 (3)	0.40537 (8)	0.79935 (7)	0.0450 (5)	0.770 (4)
O1	0.3483 (7)	0.2850 (2)	0.76836 (19)	0.0451 (11)	0.770 (4)
C1	0.3521 (12)	0.3028 (4)	0.8813 (3)	0.0361 (18)	0.770 (4)
C2	0.6090 (12)	0.3076 (8)	0.8878 (8)	0.039 (3)	0.770 (4)
H2A	0.652836	0.294448	0.931111	0.058*	0.770 (4)
H2B	0.657117	0.355274	0.879249	0.058*	0.770 (4)
H2C	0.679074	0.276143	0.857122	0.058*	0.770 (4)
C4	0.272 (2)	0.2291 (5)	0.8928 (5)	0.058 (3)	0.770 (4)
H4A	0.339000	0.198135	0.861101	0.087*	0.770 (4)
H4B	0.110117	0.227401	0.889187	0.087*	0.770 (4)
H4C	0.316890	0.214251	0.935628	0.087*	0.770 (4)
C3	0.2438 (11)	0.3521 (3)	0.9302 (3)	0.0404 (15)	0.770 (4)
H3A	0.278848	0.336261	0.973456	0.061*	0.770 (4)
H3B	0.082257	0.352166	0.924197	0.061*	0.770 (4)
H3C	0.301585	0.399154	0.924132	0.061*	0.770 (4)
C5	0.3022 (10)	0.3230 (3)	0.8119 (3)	0.0332 (12)	0.770 (4)
S1A	0.2553 (10)	0.3349 (3)	0.7649 (3)	0.055 (2)*	0.230 (4)
O1A	0.297 (2)	0.4314 (7)	0.8496 (6)	0.042 (4)*	0.230 (4)
C1A	0.355 (4)	0.3187 (10)	0.8972 (9)	0.023 (7)*	0.230 (4)
C2A	0.615 (5)	0.309 (3)	0.899 (3)	0.06 (2)*	0.230 (4)
H2AA	0.683993	0.351239	0.915726	0.089*	0.230 (4)
H2AB	0.669427	0.300462	0.855557	0.089*	0.230 (4)
H2AC	0.652259	0.269623	0.926175	0.089*	0.230 (4)
C3A	0.283 (5)	0.3463 (15)	0.9609 (11)	0.056 (8)*	0.230 (4)
H3AA	0.340463	0.316272	0.994771	0.084*	0.230 (4)
H3AB	0.120195	0.347280	0.962866	0.084*	0.230 (4)
H3AC	0.341046	0.393392	0.966587	0.084*	0.230 (4)
C4A	0.260 (6)	0.2455 (13)	0.8820 (15)	0.038 (8)*	0.230 (4)
H4AA	0.330574	0.227462	0.843296	0.058*	0.230 (4)
H4AB	0.099349	0.248796	0.875531	0.058*	0.230 (4)
H4AC	0.291241	0.214194	0.917714	0.058*	0.230 (4)
C5A	0.310 (3)	0.3695 (9)	0.8412 (8)	0.041 (5)*	0.230 (4)

### (S)-{2-[(8S,9S,10R,11S,13S,14S,17R)-11,17-Dihydroxy-10,13-dimethyl-3-oxo-2,6,7,8,9,11,12,14,15,16-decahydro-1H-cyclopenta[a]phenanthren-17-yl]-2-oxoethyl} 2,2-dimethylpropanethioate (SY20C174) Atomic displacement parameters (Å^2^)

**Table:**

	*U*^11^	*U*^22^	*U*^33^	*U*^12^	*U*^13^	*U*^23^
O2	0.057 (2)	0.066 (2)	0.0398 (19)	−0.0032 (19)	−0.0148 (17)	−0.0134 (17)
O3	0.0435 (18)	0.0303 (16)	0.0434 (18)	0.0078 (14)	−0.0008 (14)	−0.0107 (13)
O4	0.056 (2)	0.0208 (14)	0.0328 (16)	−0.0010 (14)	0.0056 (15)	0.0014 (12)
O5	0.077 (2)	0.0398 (18)	0.0297 (17)	0.0135 (18)	−0.0042 (17)	0.0032 (13)
C19	0.034 (2)	0.031 (2)	0.035 (2)	0.0098 (18)	0.0098 (18)	0.0075 (18)
C23	0.049 (3)	0.030 (2)	0.036 (2)	0.009 (2)	0.011 (2)	0.008 (2)
C10	0.029 (2)	0.025 (2)	0.043 (2)	0.0000 (17)	−0.0052 (19)	−0.0017 (19)
C26	0.033 (2)	0.030 (2)	0.031 (2)	−0.0031 (18)	0.0027 (19)	0.0025 (17)
C21	0.031 (2)	0.026 (2)	0.047 (3)	−0.0033 (18)	0.000 (2)	0.0037 (18)
C18	0.032 (2)	0.026 (2)	0.029 (2)	0.0006 (17)	0.0017 (17)	0.0055 (17)
C24	0.053 (3)	0.038 (2)	0.031 (2)	0.017 (2)	0.007 (2)	0.0035 (19)
C15	0.036 (2)	0.022 (2)	0.030 (2)	−0.0032 (18)	−0.0003 (18)	−0.0013 (16)
C8	0.040 (2)	0.032 (2)	0.044 (3)	0.005 (2)	−0.005 (2)	−0.009 (2)
C17	0.026 (2)	0.0237 (19)	0.038 (2)	0.0022 (16)	−0.0028 (17)	0.0020 (17)
C9	0.033 (2)	0.027 (2)	0.037 (2)	0.0015 (17)	−0.0020 (18)	−0.0033 (18)
C22	0.042 (2)	0.028 (2)	0.033 (2)	0.003 (2)	0.004 (2)	0.0018 (17)
C11	0.036 (2)	0.030 (2)	0.050 (3)	−0.0019 (19)	−0.007 (2)	−0.006 (2)
C20	0.036 (2)	0.031 (2)	0.047 (3)	−0.0004 (18)	0.008 (2)	0.010 (2)
C12	0.046 (3)	0.037 (2)	0.051 (3)	−0.001 (2)	−0.007 (2)	−0.015 (2)
C6	0.058 (3)	0.041 (3)	0.033 (2)	0.012 (2)	−0.002 (2)	0.000 (2)
C13	0.043 (3)	0.034 (2)	0.037 (2)	0.005 (2)	−0.006 (2)	−0.0056 (19)
C25	0.044 (2)	0.036 (2)	0.032 (2)	0.004 (2)	−0.002 (2)	0.0002 (18)
C14	0.032 (2)	0.025 (2)	0.034 (2)	−0.0018 (17)	0.0041 (17)	0.0008 (17)
C7	0.052 (3)	0.038 (2)	0.037 (3)	0.004 (2)	−0.004 (2)	−0.016 (2)
C16	0.026 (2)	0.0223 (19)	0.033 (2)	0.0016 (16)	−0.0008 (17)	0.0049 (16)
S1	0.0628 (10)	0.0413 (9)	0.0309 (8)	0.0206 (8)	0.0025 (7)	−0.0008 (6)
O1	0.052 (3)	0.042 (2)	0.042 (2)	0.007 (2)	−0.015 (2)	−0.013 (2)
C1	0.041 (4)	0.031 (4)	0.036 (4)	0.008 (3)	−0.001 (3)	−0.008 (3)
C2	0.033 (5)	0.044 (6)	0.039 (5)	0.005 (3)	−0.007 (3)	−0.007 (4)
C4	0.071 (6)	0.037 (4)	0.066 (6)	0.001 (4)	−0.013 (5)	0.012 (4)
C3	0.041 (4)	0.043 (4)	0.037 (4)	0.006 (3)	−0.001 (3)	−0.001 (3)
C5	0.032 (3)	0.030 (3)	0.037 (3)	0.001 (2)	0.000 (2)	−0.004 (3)

### (S)-{2-[(8S,9S,10R,11S,13S,14S,17R)-11,17-Dihydroxy-10,13-dimethyl-3-oxo-2,6,7,8,9,11,12,14,15,16-decahydro-1H-cyclopenta[a]phenanthren-17-yl]-2-oxoethyl} 2,2-dimethylpropanethioate (SY20C174) Geometric parameters (Å, º)

**Table:**

O2—C7	1.204 (6)	C6—H6AA	0.9900
O3—H3	0.8400	C6—H6AB	0.9900
O3—C8	1.422 (6)	C6—H6BC	0.9900
O4—H4	0.8400	C6—H6BD	0.9900
O4—C15	1.436 (5)	C6—C7	1.514 (7)
O5—C24	1.238 (5)	C6—S1	1.804 (4)
C19—C23	1.341 (6)	C6—S1A	1.807 (5)
C19—C18	1.523 (6)	C13—H13A	0.9800
C19—C20	1.491 (6)	C13—H13B	0.9800
C23—H23	0.9500	C13—H13C	0.9800
C23—C24	1.443 (7)	C25—H25A	0.9900
C10—H10	1.0000	C25—H25B	0.9900
C10—C17	1.519 (6)	C14—H14A	0.9900
C10—C9	1.542 (6)	C14—H14B	0.9900
C10—C11	1.525 (6)	C16—H16	1.0000
C26—H26A	0.9900	S1—C5	1.767 (6)
C26—H26B	0.9900	O1—C5	1.203 (6)
C26—C18	1.536 (6)	C1—C2	1.554 (9)
C26—C25	1.522 (5)	C1—C4	1.519 (11)
C21—H21A	0.9900	C1—C3	1.543 (9)
C21—H21B	0.9900	C1—C5	1.538 (9)
C21—C17	1.524 (6)	C2—H2A	0.9800
C21—C20	1.531 (6)	C2—H2B	0.9800
C18—C22	1.547 (6)	C2—H2C	0.9800
C18—C16	1.572 (6)	C4—H4A	0.9800
C24—C25	1.495 (6)	C4—H4B	0.9800
C15—H15	1.0000	C4—H4C	0.9800
C15—C14	1.543 (6)	C3—H3A	0.9800
C15—C16	1.549 (5)	C3—H3B	0.9800
C8—C9	1.565 (6)	C3—H3C	0.9800
C8—C12	1.547 (7)	S1A—C5A	1.767 (16)
C8—C7	1.543 (7)	O1A—C5A	1.209 (17)
C17—H17	1.0000	C1A—C2A	1.57 (2)
C17—C16	1.536 (6)	C1A—C3A	1.50 (2)
C9—C13	1.540 (6)	C1A—C4A	1.55 (2)
C9—C14	1.518 (6)	C1A—C5A	1.553 (19)
C22—H22A	0.9800	C2A—H2AA	0.9800
C22—H22B	0.9800	C2A—H2AB	0.9800
C22—H22C	0.9800	C2A—H2AC	0.9800
C11—H11A	0.9900	C3A—H3AA	0.9800
C11—H11B	0.9900	C3A—H3AB	0.9800
C11—C12	1.544 (7)	C3A—H3AC	0.9800
C20—H20A	0.9900	C4A—H4AA	0.9800
C20—H20B	0.9900	C4A—H4AB	0.9800
C12—H12A	0.9900	C4A—H4AC	0.9800
C12—H12B	0.9900		
			
C8—O3—H3	109.5	C7—C6—S1	113.1 (4)
C15—O4—H4	109.5	C7—C6—S1A	111.5 (4)
C23—C19—C18	122.2 (4)	S1—C6—H6AA	109.0
C23—C19—C20	121.2 (4)	S1—C6—H6AB	109.0
C20—C19—C18	116.4 (4)	S1A—C6—H6BC	109.3
C19—C23—H23	117.9	S1A—C6—H6BD	109.3
C19—C23—C24	124.3 (4)	C9—C13—H13A	109.5
C24—C23—H23	117.9	C9—C13—H13B	109.5
C17—C10—H10	106.5	C9—C13—H13C	109.5
C17—C10—C9	113.1 (3)	H13A—C13—H13B	109.5
C17—C10—C11	118.6 (4)	H13A—C13—H13C	109.5
C9—C10—H10	106.5	H13B—C13—H13C	109.5
C11—C10—H10	106.5	C26—C25—H25A	109.6
C11—C10—C9	104.9 (4)	C26—C25—H25B	109.6
H26A—C26—H26B	107.7	C24—C25—C26	110.4 (4)
C18—C26—H26A	108.8	C24—C25—H25A	109.6
C18—C26—H26B	108.8	C24—C25—H25B	109.6
C25—C26—H26A	108.8	H25A—C25—H25B	108.1
C25—C26—H26B	108.8	C15—C14—H14A	108.8
C25—C26—C18	113.8 (4)	C15—C14—H14B	108.8
H21A—C21—H21B	107.8	C9—C14—C15	113.8 (3)
C17—C21—H21A	109.0	C9—C14—H14A	108.8
C17—C21—H21B	109.0	C9—C14—H14B	108.8
C17—C21—C20	112.8 (3)	H14A—C14—H14B	107.7
C20—C21—H21A	109.0	O2—C7—C8	121.7 (5)
C20—C21—H21B	109.0	O2—C7—C6	120.7 (5)
C19—C18—C26	109.7 (3)	C6—C7—C8	117.5 (4)
C19—C18—C22	106.9 (3)	C18—C16—H16	104.1
C19—C18—C16	107.4 (3)	C15—C16—C18	114.2 (3)
C26—C18—C22	109.7 (3)	C15—C16—H16	104.1
C26—C18—C16	109.1 (3)	C17—C16—C18	113.6 (3)
C22—C18—C16	113.9 (3)	C17—C16—C15	114.9 (3)
O5—C24—C23	122.3 (4)	C17—C16—H16	104.1
O5—C24—C25	121.1 (5)	C5—S1—C6	98.4 (2)
C23—C24—C25	116.6 (4)	C4—C1—C2	110.8 (8)
O4—C15—H15	107.3	C4—C1—C3	109.7 (6)
O4—C15—C14	111.2 (3)	C4—C1—C5	109.0 (7)
O4—C15—C16	110.5 (3)	C3—C1—C2	109.0 (9)
C14—C15—H15	107.3	C5—C1—C2	105.2 (7)
C14—C15—C16	113.0 (3)	C5—C1—C3	113.1 (5)
C16—C15—H15	107.3	C1—C2—H2A	109.5
O3—C8—C9	107.5 (3)	C1—C2—H2B	109.5
O3—C8—C12	112.0 (4)	C1—C2—H2C	109.5
O3—C8—C7	109.5 (4)	H2A—C2—H2B	109.5
C12—C8—C9	103.3 (4)	H2A—C2—H2C	109.5
C7—C8—C9	112.7 (4)	H2B—C2—H2C	109.5
C7—C8—C12	111.7 (4)	C1—C4—H4A	109.5
C10—C17—C21	109.9 (3)	C1—C4—H4B	109.5
C10—C17—H17	109.5	C1—C4—H4C	109.5
C10—C17—C16	108.8 (3)	H4A—C4—H4B	109.5
C21—C17—H17	109.5	H4A—C4—H4C	109.5
C21—C17—C16	109.5 (3)	H4B—C4—H4C	109.5
C16—C17—H17	109.5	C1—C3—H3A	109.5
C10—C9—C8	100.3 (3)	C1—C3—H3B	109.5
C13—C9—C10	112.3 (4)	C1—C3—H3C	109.5
C13—C9—C8	108.7 (3)	H3A—C3—H3B	109.5
C14—C9—C10	106.9 (3)	H3A—C3—H3C	109.5
C14—C9—C8	116.2 (4)	H3B—C3—H3C	109.5
C14—C9—C13	112.0 (3)	O1—C5—S1	121.9 (4)
C18—C22—H22A	109.5	O1—C5—C1	121.4 (5)
C18—C22—H22B	109.5	C1—C5—S1	116.6 (4)
C18—C22—H22C	109.5	C5A—S1A—C6	105.7 (6)
H22A—C22—H22B	109.5	C3A—C1A—C2A	108 (3)
H22A—C22—H22C	109.5	C3A—C1A—C4A	113.4 (19)
H22B—C22—H22C	109.5	C3A—C1A—C5A	113.4 (17)
C10—C11—H11A	110.8	C4A—C1A—C2A	105 (3)
C10—C11—H11B	110.8	C5A—C1A—C2A	105 (2)
C10—C11—C12	104.6 (4)	C5A—C1A—C4A	110.7 (17)
H11A—C11—H11B	108.9	C1A—C2A—H2AA	109.5
C12—C11—H11A	110.8	C1A—C2A—H2AB	109.5
C12—C11—H11B	110.8	C1A—C2A—H2AC	109.5
C19—C20—C21	113.3 (4)	H2AA—C2A—H2AB	109.5
C19—C20—H20A	108.9	H2AA—C2A—H2AC	109.5
C19—C20—H20B	108.9	H2AB—C2A—H2AC	109.5
C21—C20—H20A	108.9	C1A—C3A—H3AA	109.5
C21—C20—H20B	108.9	C1A—C3A—H3AB	109.5
H20A—C20—H20B	107.7	C1A—C3A—H3AC	109.5
C8—C12—H12A	110.3	H3AA—C3A—H3AB	109.5
C8—C12—H12B	110.3	H3AA—C3A—H3AC	109.5
C11—C12—C8	107.0 (4)	H3AB—C3A—H3AC	109.5
C11—C12—H12A	110.3	C1A—C4A—H4AA	109.5
C11—C12—H12B	110.3	C1A—C4A—H4AB	109.5
H12A—C12—H12B	108.6	C1A—C4A—H4AC	109.5
H6AA—C6—H6AB	107.8	H4AA—C4A—H4AB	109.5
H6BC—C6—H6BD	108.0	H4AA—C4A—H4AC	109.5
C7—C6—H6AA	109.0	H4AB—C4A—H4AC	109.5
C7—C6—H6AB	109.0	O1A—C5A—S1A	119.5 (13)
C7—C6—H6BC	109.3	O1A—C5A—C1A	121.7 (15)
C7—C6—H6BD	109.3	C1A—C5A—S1A	118.6 (12)
			
O3—C8—C9—C10	78.4 (4)	C11—C10—C9—C13	−71.3 (4)
O3—C8—C9—C13	−163.7 (4)	C11—C10—C9—C14	165.5 (3)
O3—C8—C9—C14	−36.2 (5)	C20—C19—C23—C24	169.9 (4)
O3—C8—C12—C11	−92.8 (4)	C20—C19—C18—C26	169.2 (4)
O3—C8—C7—O2	−150.6 (4)	C20—C19—C18—C22	−72.0 (4)
O3—C8—C7—C6	33.2 (5)	C20—C19—C18—C16	50.7 (5)
O4—C15—C14—C9	77.5 (4)	C20—C21—C17—C10	−172.5 (3)
O4—C15—C16—C18	51.7 (4)	C20—C21—C17—C16	−53.0 (5)
O4—C15—C16—C17	−82.2 (4)	C12—C8—C9—C10	−40.1 (4)
O5—C24—C25—C26	−147.6 (4)	C12—C8—C9—C13	77.8 (4)
C19—C23—C24—O5	177.9 (4)	C12—C8—C9—C14	−154.8 (4)
C19—C23—C24—C25	−4.1 (7)	C12—C8—C7—O2	−25.9 (6)
C19—C18—C16—C15	170.8 (3)	C12—C8—C7—C6	157.8 (4)
C19—C18—C16—C17	−54.7 (4)	C6—S1—C5—O1	−8.4 (6)
C23—C19—C18—C26	−14.2 (5)	C6—S1—C5—C1	169.9 (5)
C23—C19—C18—C22	104.7 (5)	C6—S1A—C5A—O1A	4 (2)
C23—C19—C18—C16	−132.7 (4)	C6—S1A—C5A—C1A	179.1 (15)
C23—C19—C20—C21	133.4 (4)	C13—C9—C14—C15	−67.5 (5)
C23—C24—C25—C26	34.4 (6)	C25—C26—C18—C19	45.3 (5)
C10—C17—C16—C18	177.5 (3)	C25—C26—C18—C22	−71.9 (4)
C10—C17—C16—C15	−48.4 (4)	C25—C26—C18—C16	162.7 (3)
C10—C9—C14—C15	55.9 (4)	C14—C15—C16—C18	177.0 (3)
C10—C11—C12—C8	4.4 (5)	C14—C15—C16—C17	43.2 (5)
C26—C18—C16—C15	52.0 (4)	C7—C8—C9—C10	−160.8 (4)
C26—C18—C16—C17	−173.5 (3)	C7—C8—C9—C13	−42.9 (5)
C21—C17—C16—C18	57.3 (4)	C7—C8—C9—C14	84.5 (5)
C21—C17—C16—C15	−168.5 (3)	C7—C8—C12—C11	143.9 (4)
C18—C19—C23—C24	−6.6 (7)	C7—C6—S1—C5	−90.2 (4)
C18—C19—C20—C21	−49.9 (5)	C7—C6—S1A—C5A	81.0 (8)
C18—C26—C25—C24	−56.1 (5)	C16—C15—C14—C9	−47.5 (5)
C8—C9—C14—C15	166.8 (3)	S1—C6—C7—O2	30.1 (6)
C17—C10—C9—C8	174.8 (3)	S1—C6—C7—C8	−153.6 (3)
C17—C10—C9—C13	59.5 (5)	C2—C1—C5—S1	−106.9 (8)
C17—C10—C9—C14	−63.7 (4)	C2—C1—C5—O1	71.4 (10)
C17—C10—C11—C12	−158.0 (4)	C4—C1—C5—S1	134.2 (6)
C17—C21—C20—C19	49.7 (5)	C4—C1—C5—O1	−47.5 (9)
C9—C10—C17—C21	179.7 (3)	C3—C1—C5—S1	11.9 (7)
C9—C10—C17—C16	59.8 (4)	C3—C1—C5—O1	−169.8 (6)
C9—C10—C11—C12	−30.4 (4)	S1A—C6—C7—O2	−27.2 (6)
C9—C8—C12—C11	22.6 (5)	S1A—C6—C7—C8	149.1 (4)
C9—C8—C7—O2	89.9 (5)	C2A—C1A—C5A—S1A	92 (3)
C9—C8—C7—C6	−86.4 (5)	C2A—C1A—C5A—O1A	−93 (3)
C22—C18—C16—C15	−70.9 (4)	C3A—C1A—C5A—S1A	−149.7 (18)
C22—C18—C16—C17	63.5 (4)	C3A—C1A—C5A—O1A	25 (3)
C11—C10—C17—C21	−56.7 (5)	C4A—C1A—C5A—S1A	−21 (2)
C11—C10—C17—C16	−176.6 (4)	C4A—C1A—C5A—O1A	154 (2)
C11—C10—C9—C8	44.0 (4)		

### (S)-{2-[(8S,9S,10R,11S,13S,14S,17R)-11,17-Dihydroxy-10,13-dimethyl-3-oxo-2,6,7,8,9,11,12,14,15,16-decahydro-1H-cyclopenta[a]phenanthren-17-yl]-2-oxoethyl} 2,2-dimethylpropanethioate (SY20C174) Hydrogen-bond geometry (Å, º)

**Table:**

*D*—H···*A*	*D*—H	H···*A*	*D*···*A*	*D*—H···*A*
O3—H3···O5^i^	0.84	1.96	2.802 (4)	175

Symmetry code: (i) −*x*+1/2, −*y*+1, *z*+1/2.
